# Autophagy Induction by Trichodermic Acid Attenuates Endoplasmic Reticulum Stress-Mediated Apoptosis in Colon Cancer Cells

**DOI:** 10.3390/ijms22115566

**Published:** 2021-05-25

**Authors:** Junyan Qu, Cheng Zeng, Tingting Zou, Xu Chen, Xiaolong Yang, Zhenghong Lin

**Affiliations:** 1 School of Life Sciences, Chongqing University, Chongqing 401331, China; qujunyan1229@yeah.net (J.Q.); cqzengcheng@163.com (C.Z.); tingtzou1997@163.com (T.Z.); chenxu_2021@163.com (X.C.); 2 The Modernization Engineering Technology Research Center of Ethnic Minority Medicine of Hubei Province, School of Pharmaceutical Sciences, South-Central University for Nationalities, Wuhan 430074, China

**Keywords:** TDA, ER stress, apoptosis, autophagy, colorectal cancer

## Abstract

Colorectal cancer (CRC) is the third leading malignant tumor in the world, which has high morbidity and mortality. In this study we found that trichodermic acid (TDA), a secondary metabolite isolated from the plant endophytic fungus *Penicillium ochrochloronthe* with a variety of biological and pharmacological activities, exhibited the antitumor effects on colorectal cancer cells in vitro and in vivo. Our results showed that TDA inhibited the proliferation of colon cancer cells in a dose-dependent manner. TDA induces sustained endoplasmic reticulum stress, which triggers apoptosis through IRE1α/XBP1 and PERK/ATF4/CHOP pathways. In addition, we found that TDA mediated endoplasmic reticulum stress also induces autophagy as a protective mechanism. Moreover, combined treatment of TDA with autophagy inhibitors significantly enhanced its anticancer effect. In conclusion, our results indicated that TDA can induce ER stress and autophagy mediated apoptosis, suggesting that targeting ER stress and autophagy may be an effective strategy for the treatment of CRC.

## 1. Introduction

Cancer increasingly becomes the main cause of deaths around the world and the most important obstacle to improving life expectancy in the 21st century [1]. Colorectal cancer (CRC) is the third leading cancer among men and the second among women worldwide [2], with high incidence and low survival rate [3]. The incidence of CRC continues to increase at an alarming rate due to changes in modern urban lifestyles. The aging population and eating habits, obesity, lack of physical exercise, smoking and all the other unfavorable risk factors increase the risk of CRC [4]. At present, about 1.4 million of new patients are diagnosed with CRC every year, and the total annual death is about 700,000. With the continuous development of CRC in developing countries, it is estimated that by 2030, the global burden of CRC will increase by 60% to more than 2.2 million new cases and 1.1 million cancer deaths [3,5]. Currently, radiotherapy, surgery, immunotherapy and chemotherapy are commonly used methods for the treatment of CRC, but the survival rate of patients using these methods alone is not very satisfactory, and the 5-year survival rate of advanced patients is still less than 15% [4,6,7]. In addition, many drugs used have serious side effects, including multidrug resistance, gastrointestinal toxicity and blood level decline, liver toxicity, which directly reduce the success rate of treatment [8,9,10,11]. For example, the incidence of thrombocytopenia in patients who received oxaliplatin in chemotherapy regimens was much higher than in patients who did not receive oxaliplatin, and the use of penfluorouracil often resulted in intestinal mucositis [10,11]. Therefore, the development of new and effective antitumor drugs is still an important strategy for the treatment of CRC.

Trichodermic acid (TDA) is a natural product isolated from the solid-substrate fermentation culture of *Penicillium ochrochloronthe* associated the roots of *Taxus media* by our coworkers [12]. It has been reported that TDA has antibacterial, antifungal and anti-inflammatory biological activities, and has obvious inhibitory ability on various cancer cells such as lung cancer, liver cancer, stomach cancer and colon cancer [12,13], showing its good potential to become a new anticancer drug. AMF-26, a derivative of TDA [14], has been shown to have good anticancer effects in many studies [15,16,17,18,19,20]. Among them, the disruption of endoplasmic reticulum to Golgi transport is the main mechanism of AMF-26′s anticancer activity. This suggests that TDA may achieve its inhibitory effect on tumor cells by acting on the function of endoplasmic reticulum. In recent years, endoplasmic reticulum stress is also a hot topic in cancer treatment, which may be the potential targeting pathway of TDA.

Endoplasmic reticulum stress (ER stress) is a highly conservative cellular response. Proteins undergo specific signal pathways to fold, aggregate and transport in the endoplasmic reticulum, maintaining the dynamic balance of environmental material levels in the cell [21,22,23]. However, when some external stimuli break the balance of the ER, the protein synthesis signal will be interrupted, causing the accumulation of unfolded and misfolded proteins in the ER, disturbing the homeostasis and ultimately leading to ER stress [24,25,26]. Appropriate ER stress is conducive to the recovery of intracellular calcium ion and protein processing, and enhances the ability of cells to withstand stress stimulation. Severe and continuous ER stress often triggers cell apoptosis [27,28]. ER stress will activate a series of cellular regulatory mechanisms, the most typical of which is the unfolded protein response (UPR) [29]. UPR relieves stress in the early stage and triggers apoptosis when the homeostasis fails to maintain for a long time [30,31].

UPR involves endoplasmic reticulum molecular chaperones (HSPA5/GRP78), endoplasmic reticulum stress sensor proteins (PERK, IRE1α and ATF6) and their downstream signaling pathways [32,33]. Under normal conditions, these three transmembrane sensor proteins (PERK, IRE1α and ATF6) bind to the ER molecular chaperone HSPA5 (also known as GRP78) and are in an inactive state [34]. In the process of ER stress, PERK dissociates from HSPA5 and activates ATF4, thereby promoting the transcription of genes related to cell survival and the proapoptotic factor CHOP [35]. After IRE1α is activated, it can cut the mRNA encoding XBP1 to generate active transcription factor XBP1s (spliced XBP1, XBP1s). After XBP1s is translocated to the nucleus, it can upregulate the expression of genes related to ER stress [36]. IRE1α can also induce cell apoptosis through TRAF2-activated JNK pathway and caspase-12 mediated signaling pathway [37]. After the dissociation of ATF6 and HSPA5, ATF6 will be transported from the ER to the Golgi to be cleaved and activated. The activated ATF6 enters the nucleus and alleviates ER stress from multiple levels by initiating a series of gene transcripts [38]. In addition to the two classic apoptosis pathways, the mitochondrial pathway and the death receptor pathway, apoptosis induced by ER stress is a common apoptotic pathway newly discovered in recent years. Additionally, it has been proved in many studies that ER stress-induced apoptosis is the molecular mechanism of many anticancer drugs [39]. Tunicamycin is an ER stress inducer, which can inhibit protein glycosylation and maturation in eukaryotes [40]. According to reports, tunicamycin reduces the proliferation, invasion and chemotaxis activities of breast tumor cells in nude mice [41]. In addition, Tunicamycin promotes the apoptosis of colon cancer cells through the AKT/mTOR signaling pathway and inhibits tumor growth in vivo and in vitro [42]. Combination therapy with tunicamycin, BAY 11-7082 and naringin induce ER stress and induce apoptosis of HT29 colon cancer cells through the PERK/eIF2α/ATF4/CHOP pathway [43]. Cucurbitacin I can activate two of the three ERS pathways, IRE1α and PERK and CHOP, thereby inducing apoptosis in SKOV3 and PANC-1 cells [44]. Recently, ER stress has also been reported as a major mechanism that celecoxib can induce cell death in cancer [45]. Therefore, activating the proapoptotic function of UPR through long-term or severe ER stress is considered to be an attractive cancer treatment strategy [39,46].

Meanwhile, the continuous stress of the endoplasmic reticulum may lead to autophagy. Autophagy is a self-degradation process that is important for balancing energy sources during the critical period of development and in response to nutritional stress [47,48]. During autophagy, the cytoplasmic contents of cells are sequestered in double-membrane vacuoles called autophagosomes, and then transported to lysosomes for degradation [49]. It is generally believed that under hypoxia, nutritional deficiencies, and anti-cancer treatments (such as chemotherapy and radiation therapy), cancer cells can continue to survive through autophagy [50,51,52]. The results of early clinical trials using hydroxychloroquine to treat cancer indicate that autophagy inhibition may be a promising method for the treatment of advanced cancer [53]. Studies have shown that autophagy has a dual role in promoting and inhibiting tumors in CRC [54,55,56], but its mechanism is still unclear. This difference in opposing effects is usually due to differences in the cells and tumor models used. This suggests that the role of autophagy in regulating cell death is highly dependent on cell type and stimulation conditions [57,58]. Therefore, further research on autophagy will reveal its potential therapeutic effects in diseases, and the regulation of autophagy may become a new direction for the drug development of cancer therapy [59,60].

In this study, we found that TDA can significantly inhibit the growth of colon cancer cells both in vitro and in vivo. In vitro experiments have shown that TDA induces ER stress-mediated apoptosis by upregulating the IRE1α and PERK pathways and activating the subsequent CHOP and caspases proteins. At the same time, ER stress induces autophagy as a cytoprotective mechanism to reduce ER pressure. Therefore, treatment combined with autophagy inhibitors can enhance the anti-CRC effect of TDA both in vivo and in vitro. Overall, our results provide new insights into the molecular basis of CRC treatment and demonstrate the potential of TDA to treat cancer.

## 2. Results

### 2.1. TDA Inhibits Colon Cancer Cell Viability In Vitro

**Figure 1:**
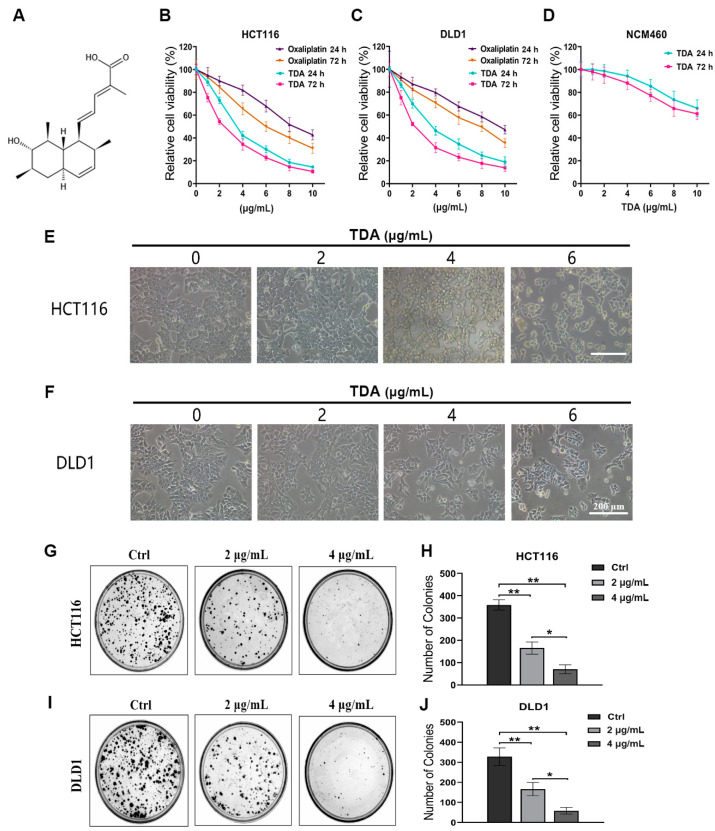
TDA inhibits cell viability in colon cancer cells. (**A**) The chemical structure of TDA. (**B**,**C**) HCT116 and DLD1 cells were treated with TDA or oxaliplatin (0, 1, 2, 4, 6, 8 and 10 μg/mL) for 24 or 72 h, respectively. Cell viability was measured by CCK-8 assay. The absorbance was measured at 450 nm. (**D**) NCM460 cells were treated with TDA (0, 1, 2, 4, 6, 8 and 10 μg/mL) for 24 or 72 h, respectively. Cell viability was measured by CCK-8 assay. The absorbance was measured at 450 nm. (**E**,**F**) Morphological observation of colon cancer cells treated with TDA at indicated concentration. (**G**–**J**) Colony formation assay of HCT116 and DLD1 cells treated with the indicated concentrations of TDA. Representative images (**G**,**I**) and quantification of colonies (**H**,**J**) was shown. Data are presented as the mean ± SD from at least three separate experiments. * *p* < 0.05; ** *p* < 0.01.

Trichodermic acid (TDA) is a natural product isolated from *Penicillium ochrochloronthe*, an endophytic fungus derived from *Taxus media* [12]. The chemical structure of TDA is shown in Figure 1A and was characterized by comparison of its spectroscopic data (see Figures S1–S3) with those reported in the literature [13]. In previous studies, TDA has shown promising anticancer therapeutic potential. To determine the effect of TDA on colon cancer, we first evaluated the cell growth of two colon cancer cell lines (HCT116 and DLD1) and human colon mucosal epithelial cell line (NCM460) after TDA treatment. As revealed in Figure 1B–D, TDA inhibited the growth of colon cancer cells in a dose-dependent and time-dependent manner. The viability of HCT116 and DLD1 cells decreased rapidly in the presence of 1−6 μg/mL TDA, while NCM460 cells showed higher tolerance to TDA. At the same time, oxaliplatin, the standardized drug for colon cancer, was used as a comparison. As shown in Figure 1B,C, compared with oxaliplatin, colon cancer cells were more sensitive to the toxic response of TDA, which also proved its anticancer effect. According to the inhibitory ratio, we calculated that half-maximal inhibitory concentration (IC_50_) of TDA toward HCT116 and DLD1 cells at 24 h were 3.410 and 3.866 µg/mL, respectively. Based on the IC_50_ value, we choose 0, 2, 4 and 6 μg/mL of TDA to conduct the following experiments. Consistently, the proliferation of colon cancer cells was significantly inhibited under TDA treatment, as evidenced by retarded cell growth (Figure 1E,F) and reduced colony formation (Figure 1G–J). At the same time, the proliferation of colon cancer cells decreased obviously with TDA treatment in a dose-dependent manner. Together, these results indicated that TDA exhibits a considerable anticancer effect in colon cancer cells in vitro.

### 2.2. TDA Treatment Induces Cell Cycle Arrest and Apoptosis in Colon Cancer Cells

**Figure 2:**
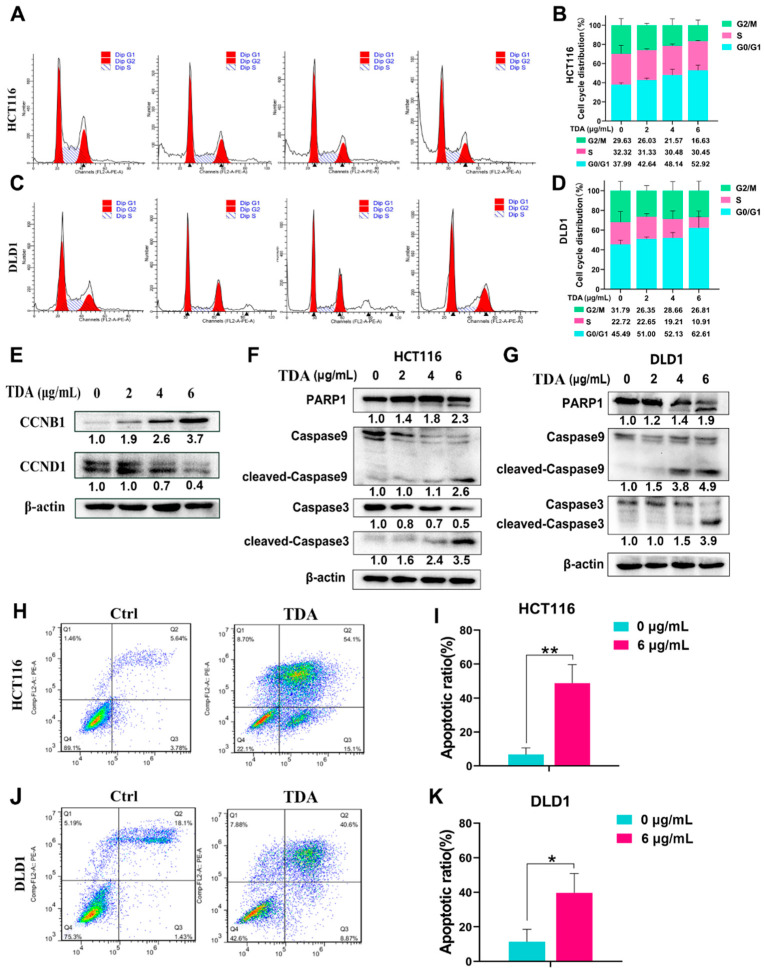
Flow cytometric analysis of colon cancer cells with TDA treatment. (**A**–**D**) Flow cytometric analysis of cell cycle in HCT116 and DLD1 cells upon TDA treatment as indicated concentration for 24 h was performed. (**E**) The expression of cyclin B1 and cyclin D1 in HCT116 cells with TDA treatment as indicated concentration for 24 h was detected by immunoblotting analysis. Beta-actin was used as internal control. (**F**,**G**) The protein expression levels of PARP1, cleaved PARP1, Caspase 9, cleaved Caspase 9, Caspase 3 and cleaved Caspase 3 in colon cancer cells treated with the indicated concentrations of TDA for 24 h were detected by Western blot. (**H**–**K**) Flow cytometric analysis of apoptosis in HCT116 and DLD1 cells upon TDA (6 μg/mL) treatment was performed for 24 h. (**H**,**J**). The percentage of apoptotic HCT116 and DLD1 cells treated with TDA is shown (**I**,**K**). Western blot results were quantified using Image J software, and the results were numerically marked below each band. Data are presented as the mean ± SD from at least three separate experiments. * *p* < 0.05; ** *p* < 0.01.

Cell cycle arrest plays a key role in antitumor therapy, so we tested whether TDA treatment can induce cell cycle changes in colon cancer cells. Flow cytometry analysis showed that after TDA treatment, the proportion of cells in G0/G1 phase increased by 14.93% (HCT116) and 17.12% (DLD1) on average, while the total proportion of cells in the G2/M phase and S phase showed a significant downward trend (Figure 2A–D). Similarly, Western blot results showed an increase in CCNB1 level and a decrease in CCND1 level (Figure 2E), indicating that cell cycle was arrested in the G0/G1 phase with the TDA treatment. However, unlike TDA, the cell cycle of colon cancer under the influence of the standard drug oxaliplatin presents G2/M phase arrest (Figure S4), suggesting that TDA may have different therapeutic targets from oxaliplatin.

To examine whether TDA induces apoptosis of colon cancer cells, we first examined the proapoptotic effect of TDA by examining the expression of apoptosis related genes. Using Western blotting, we measured the activation of apoptotic proteins caspase-9, caspase-3 and PARP1. In HCT116 and DLD1, we found that the cleavage levels of caspase-9, -3 and PARP1 in cells treated with TDA were observably higher than those in untreated cells (Figure 2F,G). Next, we performed flow cytometry analysis by phosphatidylserine translocation using FITC-annexin V and propidium iodide (PI) double-staining. Consistent with the above results, our data showed a 30−40% increase in the percentage of apoptotic cells in the TDA treatment group, most of which occurred in the late apoptotic region (Figure 2H–K). Taken together, these results suggested that TDA can induce the apoptosis of colon cancer cells.

### 2.3. Transcriptome Analysis Identified the Apoptotic- and Autophagy-Related Genes Contributive to the TDA-Induced Cell Death

**Figure 3:**
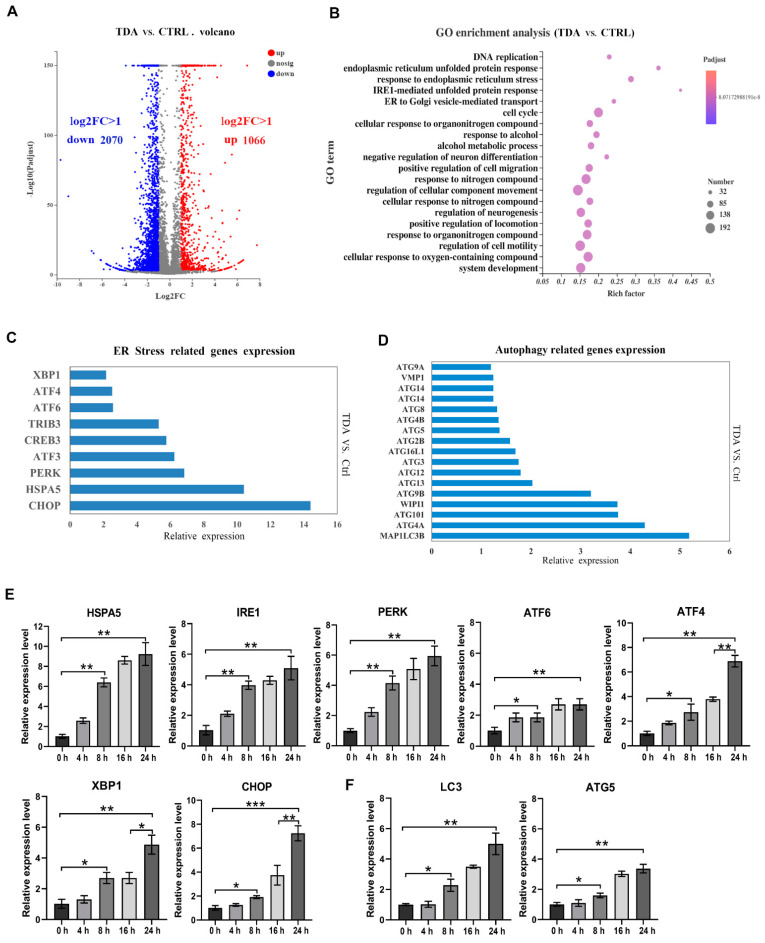
Transcriptome analysis of TDA-treated HCT116 cells. (**A**) The number of upregulated and downregulated differentially expressed genes in the transcriptome upon TDA (4 μg/mL) for 24 h treatment is shown in the volcano map. (**B**) GO enrichment analysis of the signaling pathways of differentially expressed genes in the transcriptome. (**C**) The relative expression of genes related to ER stress pathway in transcriptome analysis was shown. (**D**) The relative expression of autophagy related genes in transcriptome analysis was shown. (**E**,**F**) The mRNA level of ER stress related genes (**E**) and autophagy related genes (**F**) were determined by real-time quantitative PCR after treatment with 6 μg/mL TDA for indicated time. Data are presented as the mean ± SD from three separate experiments. * *p* < 0.05; ** *p* < 0.01; *** *p* < 0.001.

In order to explore the underlying mechanism of TDA inducing apoptosis of colon cancer cells, we used RNA sequencing (RNA-seq) to study the transcriptome of HCT116 cells treated with TDA (4 μg/mL) for 24 h in comparison with untreated control cells. The analysis identified 3136 differentially expressed genes (*p* < 0.05, fold change > 2), of which 1066 were upregulated and 2070 were downregulated (Figure 3A). Differential gene enrichment analysis showed that TDA affected cellular components, biological processes and molecular functions by regulating 3136 genes in various pathways. The top 20 enriched by Gene Ontology have been listed (Figure 3B). Among them, endoplasmic reticulum unfolded protein response, response to endoplasmic reticulum stress and IRE1-mediated unfolded protein response occupy the top ones. At the same time, we observed that the transcription level of the ER stress related genes (HSPA5, PERK, CHOP, XBP1, etc.) significantly increased in the TDA treated cells (Figure 3C). In conclusion, we speculated that TDA induced ER stress in colon cancer cells. Accumulation of misfolded proteins can cause ER stress, which in turn induces an adaptive intracellular response known as UPR. However, when UPR cannot completely relieve ER stress, cells undergo apoptosis or autophagy. On the other hand, we found that autophagy-related genes (ATG and MAPILC3B) were also significantly upregulated in the TDA treatment group (Figure 3D), indicating that autophagy may be also involved in the toxic effect of TDA on colon cancer cells.

Next, we verified the results of transcriptome analysis by RT-PCR, and the mRNA levels of HSPA5, IRE1α, XBP1, PERK, ATF4 and CHOP all increased visibly in a time-dependent manner. As expected, mRNA levels of HSPA5, PERK and IRE1α had increased observably before 16 h, while ATF4 and CHOP showed a more significant increase at 24 h, which was consistent with the action pattern of ER stress (Figure 3E). Moreover, LC3 and ATG5 expression were upregulated upon TDA treatment (Figure 3F). In summary, we hypothesized that TDA-induced apoptosis is achieved through the ER stress pathway, and autophagy is also involved in the process.

### 2.4. TDA Induced Apoptosis through IRE1α and PERK Pathways

**Figure 4:**
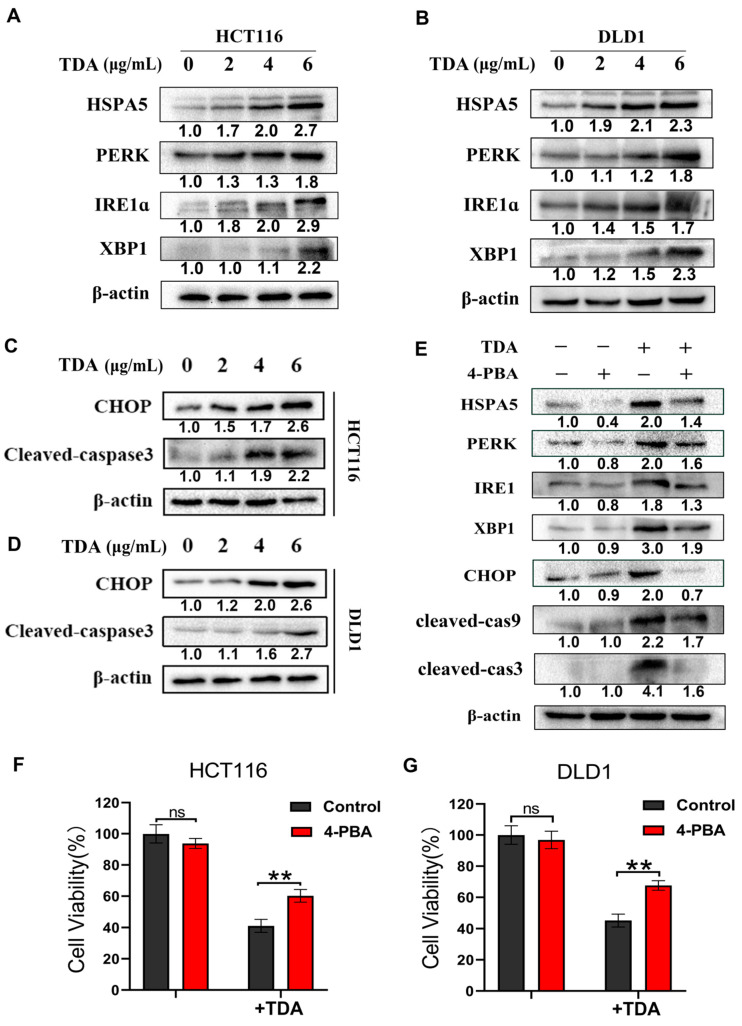
TDA activates ER stress response to induce apoptosis in colon cancer cells. (**A**,**B**) Western blot detection of the expression of HSPA5, PERK, IRE1α and XBP1 in HCT116 and DLD1 cells treated with the indicated concentrations of TDA for 24 h. (**C**,**D**) The protein expression levels of CHOP and cleaved Caspase 3 in TDA-treated HCT116 (**C**) or DLD1 (**D**) cells were detected by Western blot. (**E**) Western blot detection of the expression of HSPA5, PERK, IRE1α and CHOP as well as cleaved Caspase 9 (cleaved-cas9), cleaved Caspase 3 (cleaved-cas3) in HCT116 cells treated with or without 6 μg/mL TDA in the presence or absence of 2 mM 4-phenylbutyrate (4-PBA) for 24 h. (**F**,**G**) Cell viability in HCT116 or DLD1cells treated with 4-PBA or TDA alone or together was measured by CCK-8 assay. Western blot results were quantified using Image J software, and the results were numerically marked below each band. Data are presented as the mean ± SD from three separate experiments. ns, no statistical significance; ** *p* < 0.01.

The ER is a highly dynamic organelle in eukaryotic cells. Many studies have shown that once UPRs (PERK, ATF6 and IRE1α) pathway are activated, they initiate early adaptive responses by increasing HSPA5 expression, thus regulating the transcription and translation of a series of genes. However, with prolonged stress, additional responses were activated, including caspase 12/9/3 and ATF4/CHOP, which promoted apoptosis. To determine whether TDA activates endoplasmic reticulum stress in colon cancer cells, we examined the expression of PERK, IRE1α, XBP1 and HSPA5 using Western blot. We observed elevated levels of these proteins after TDA treatment in a dose-dependent manner, indicating an activation of ER stress (Figure 4A,B). Meanwhile, we found that CHOP and cleaved-caspase 3 also increased after TDA treatment, further suggesting that TDA-induced ER stress leads to cell apoptosis (Figure 4C,D).

To evaluate whether ER stress plays a major role in TDA-induced apoptosis, HCT116 cells were treated with TDA combined with an ER stress inhibitor, 4-phenylbutyrate (4-PBA). As shown in Figure 4E, 4-PBA treatment significantly reduced the expression of HSPA5, PERK and IRE1 induced by TDA, while the protein levels of apoptosis-related proteins CHOP and cleaved caspase 3 also decreased under the combined treatment of 4-PBA. Importantly, by measuring cell viability by CCK-8, we observed that in the cells treated with TDA combined with 4-PBA, cell viability was increased compared with TDA alone, indicating that inhibition of endoplasmic reticulum stress reduced apoptotic proportion of the cells (Figure 4F,G). In summary, these results indicated that the endoplasmic reticulum stress pathway dominated the TDA-induced apoptosis of colon cancer cells.

### 2.5. Endoplasmic Reticulum Stress Induces Protective Autophagy

**Figure 5:**
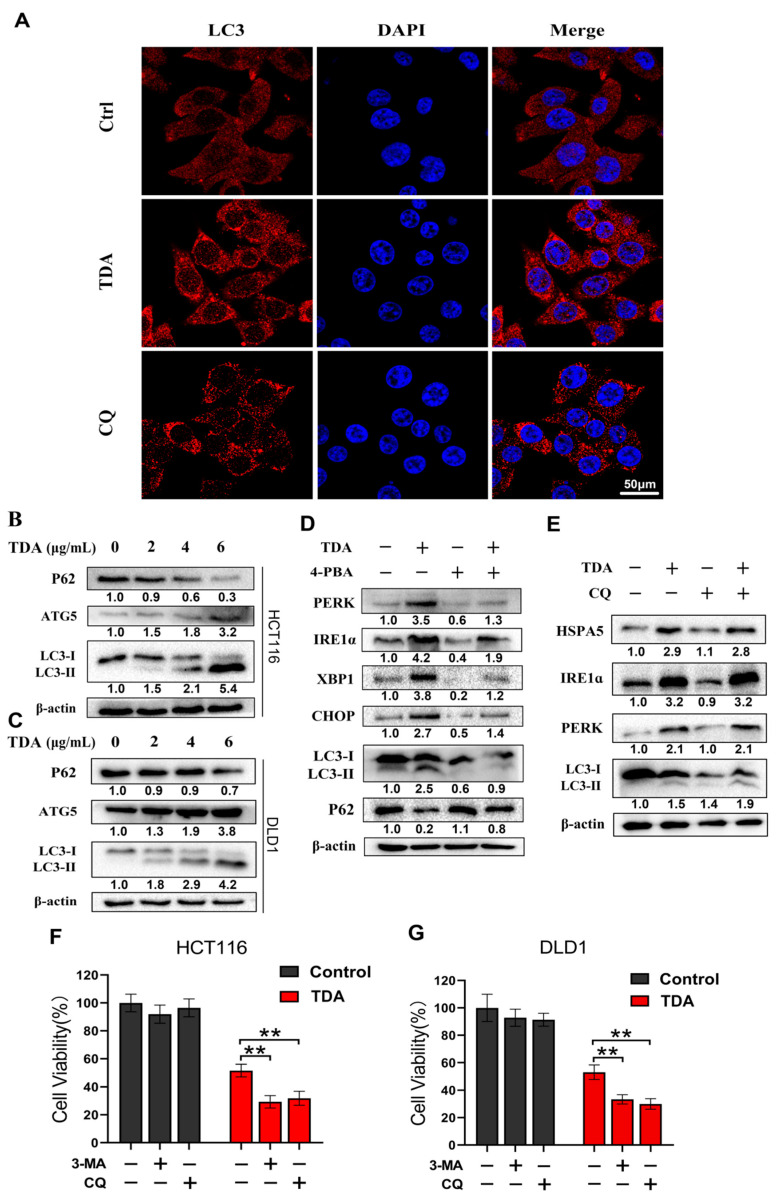
TDA induces autophagy in colon cancer cells. (**A**) Analysis of autophagy induction by immunofluorescence staining of LC3B in HCT116 cells, which were treated with TDA (3 μg/mL) for 24 h. CQ (10 μM) was treated for 24 h as a positive control. (**B**,**C**) The protein expression levels of P62, ATG5 and LC3 in TDA-treated HCT116 or DLD1 cells were detected by Western blot. (**D**) Immunoblotting of PERK, IRE1α, LC3 and P62 in HCT116 cells treated with TDA, 4-PBA alone or together as indicated for 24 h. (**E**) Immunoblotting of HSPA5, PERK, IRE1α and LC3B in HCT116 cells treated with TDA, CQ alone or together as indicated for 24 h. (**F**,**G**) HCT116 and DLD1 cells were treated with TDA alone or combination with autophagy inhibitors 3-MA or CQ as indicated. Cell viability was measured by CCK-8 assay. Cells were pretreated with 3-MA (1 mM) or CQ (10 μM) for 4 h, followed by TDA (6 μg/mL) treatment for 24 h. Data are presented as mean ± SD. ** *p* < 0.01. Western blot results were quantified using software Image J v1.8.0 (National Institutes of Health, USA) and the results were numerically marked below each band.

Autophagy is an evolutionarily conserved mechanism that maintains cell homeostasis. More and more evidence suggest that ER stress-induced UPR activation promotes autophagy to coordinate ER homeostasis. We also observed upregulation of autophagy-related genes in transcriptome data. Therefore, to clarify whether TDA induces autophagy in colon cancer cells, we analyzed the changes of autophagy flux with or without TDA treatment from three aspects. Firstly, fluorescence microscopy evaluation showed that the number of autophagosomes in TDA treated cells increased after 24 h treatment (Figure 5A). Secondly, we found that TDA induces the conversion of LC3-I to lipidated LC3-II (an established autophagosome marker) (Figure 5B,C). Thirdly, we evaluated the expression of autophagy-related proteins ATG5 and P62 (Figure 5B,C). ATG5 is a key protein involved in the formation of autophagosome, and P62 is a marker of autophagy degradation. When autophagy is triggered, P62 is decreased and when autophagy is inhibited, P62 accumulates. The results showed that TDA significantly reduced P62 levels. In contrast, TDA increased the expression of ATG5 (Figure 5B,C). Taken together, these data suggest that TDA induces autophagy in colon cancer cells.

To investigate whether autophagy induction is an adaptive process in response to ER stress, we examined the expression of autophagy-related proteins in HCT116 cells treated with TDA alone and in combination with 4-PBA. As shown in Figure 5D, 4-PBA inhibited TDA-induced upregulation of autophagy-related proteins. In contrast, the autophagy inhibitor chloroquine (CQ) had no significant effect on the expression of ER stress markers in TDA-treated cells (Figure 5E). These data indicated that TDA-induced ER stress is an upstream event of autophagy.

Abnormal autophagy plays a dual role in promoting cancer cell survival and cell death in colon cancer. To elucidate the role of TDA in inducing autophagy in colon cancer cells, we treated cells with TDA alone or in combination with autophagy inhibitor 3-methyladenine (3-MA) or CQ. As shown in Figure 5F,G, an increase in cell toxicity were observed following CQ or 3-MA treatment in TDA-treated colon cancer cells. This suggested that TDA-induced autophagy is protective but not harmful in colon cancer cells. 

### 2.6. Treatment of TDA Retards the Growth of Transplanted Tumors

**Figure 6:**
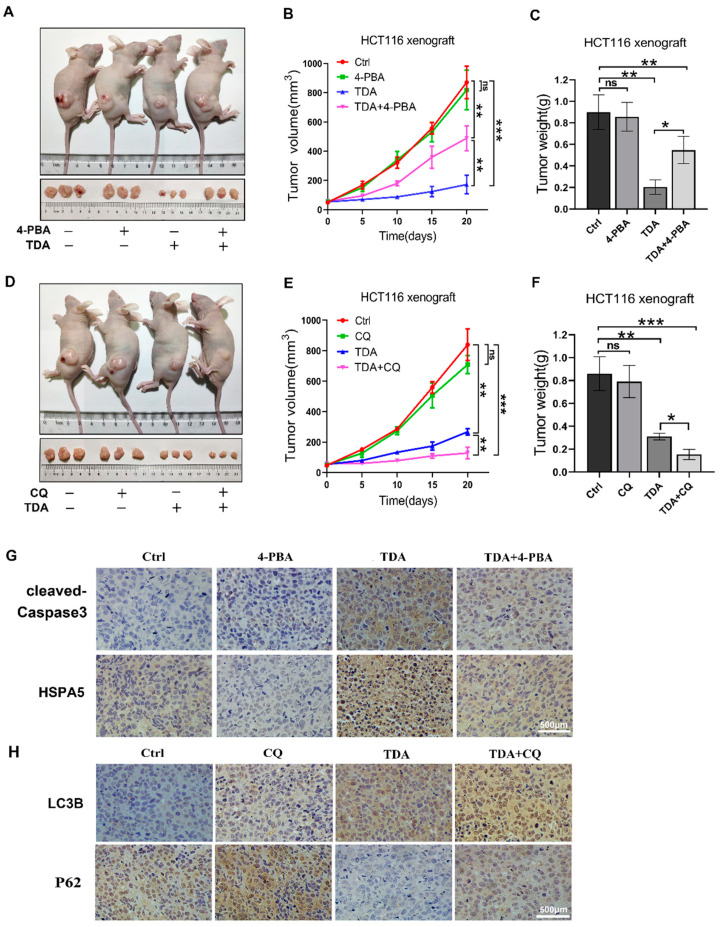
TDA inhibits the tumor growth in vivo. HCT116 cells were injected subcutaneously into 6-week-old nude mice. (**A**–**C**) When the tumor volumes reached 100 mm^3^, mice were treated with TDA alone or combination with 4-PBA as indicated. Images (**A**) and tumor weight (**B**) and volumes measured every 5 days (**C**) were shown. (**D**–**F**) When the tumor volumes reached 100 mm^3^, mice were treated with TDA alone or combination with CQ as indicated. Images (**D**) and tumor weight (**E**) and volumes measured every 5 days (**F**) were shown. (**G**,**H**) Immunohistochemical staining analysis was performed to detect the expression of cleaved Caspase3, HSPA5 (**G**), LC3B or P62 (**H**). Data are presented as mean ± SD. ns, no statistical significance; * *p* < 0.05; ** *p* < 0.01; *** *p* < 0.001.

In order to further test the anticancer effect of TDA in vivo, we subcutaneously inoculated human colon cancer HCT116 cells into 6-week-old nude mice to establish a xenograft tumor model. Compared with the control group, xenograft tumors treated with TDA alone showed a significant reduction in volume, growth rate and weight (Figure 6A–C). Notably, the efficacy of TDA was reduced in combination with 4-PBA compared to TDA alone (Figure 6A–C). In contrast, the size, growth rate and weight of xenograft tumors treated with TDA combined with CQ were further reduced (Figure 6D–F). In addition, obvious ER stress and apoptosis were observed in mouse tumors treated with TDA, as evidenced by increased HSPA5 and cleaved-caspase-3 levels in immunohistochemistry, while single treatment of TDA displayed more robust cleaved-caspase 3 staining (Figure 6G), which further proved that the apoptosis induced by TDA is dominated by the ER stress pathway. We also observed an increase in LC3B expression and a decrease in P62 expression by immunohistochemical staining (Figure 6H). These results suggest that CQ enhances the antitumor effect of TDA by inhibiting autophagy of colon cancer cells.

## 3. Discussion

CRC is still one of the notorious cancers at present [4]. Although the current research made significant progress in the mechanism understanding of tumorigenesis and developed multiple treatments such as surgery, radiotherapy and chemotherapy, the 5-year survival rate of advanced stage patients is still less than 15% [6,7]. This low survival rate is due to late diagnosis and resistance to current chemotherapy [9]. Therefore, new and effective treatment approaches are still essential and urgent.

In recent years, natural products have aroused people’s interest in the development of new anticancer therapeutic drugs [61,62,63], because most of them show good potential of medicinal significance, which can be used as alternative drugs for the treatment of various diseases (including cancer) with few side effects relatively [64,65]. TDA is a newly discovered octahydronaphthalene derivative, isolated from the secondary metabolites of plant endophytic fungi, and has shown anticancer activity in a variety of cancers [12,13]. However, until now, the anticancer mechanism of TDA remains unclear. Therefore, in this study, we explored the antitumor activity and molecular mechanism of TDA in colon cancer. At first, we found that genes related to ER stress pathway and autophagy pathway were significantly activated in TDA treated cells through transcriptome analysis. In subsequent studies, we mainly analyzed the death-promoting mechanism of TDA from these two aspects.

Recently, ER stress and unfolded protein response (UPR) have attracted extensive attention as targets of tumor therapy [39,66]. The ER is responsible for the correct folding and post-translational modification of proteins, calcium storage and lipid synthesis [22,23]. The disturbance of ER homeostasis usually leads to ER stress, and UPR is activated to relieve ER pressure. PERK, IRE1α and ATF6 are sensors of ER stress and markers of UPR. They can promote transcription of genes responsible for maintaining ER homeostasis, but under certain conditions, they may extend ER stress and eventually lead to the proapoptotic pathway [24,28,46]. In our study, the protein expression levels of PERK, IRE1α and XBP1 were significantly increased after TDA treatment. Meanwhile, the apoptotic marker CHOP and caspase protein were also activated, indicating that the proapoptotic pathway of ER stress had been triggered. In addition, the combined treatment of ER stress inhibitor 4-PBA and TDA attenuated the therapeutic effect of TDA, further demonstrating that the activation of UPR dominates the apoptosis induced by TDA. According to the survival curve of colon cancer patients, patients with high expression of ER stress-related proteins such as HSPA5, IRE1α and PERK have a higher survival time than those with low expression, suggesting that UPR activation may have a potential tumor suppressive effect, especially when ER stress intensity is high and persistent.

Due to the harsh living environment of tumor cells, most malignant tumor cells themselves have slight chronic ER stress, and ER chaperone HSPA5 is often highly expressed in tumor cells [67]. ER stress-induced cell death is controlled by many factors, including calcium ions, reactive oxygen species and proapoptotic proteins [68]. Among them, CHOP is a key protein in this process [69]. Despite tumor cells have mild ER stress, the expression of CHOP is still at a low level. However, levels of CHOP and other apoptotic proteins were greatly increased in severe ER stress. The extent and duration of this increase is a determining factor in the cell fate [70,71]. Therefore, it may be an opportunity for therapeutic interventions to further aggravate the existing stress conditions in tumor cells by appropriate drugs, forcing the ER stress response into a proapoptotic mode. At present, many drugs have been used to induce apoptosis of the ER stress pathway. These drugs are also called ER stress aggravating agents (ERSA) [72], such as drugs perturbing calcium levels (thapsigargin, ionomycin and A-23187) [73,74], impairment of protein folding (2-deoxyglucose, tunicamycin and geldanamycin) [73,75,76] and inhibiting protein trafficking (brefeldin A and monensin) [77,78]. Among them, thapsigargin, tunicamycin and brefeldin A are the most widely used [39]. Current research is trying to use these compounds in clinical anticancer applications, but unfortunately, most of the drugs have been shown to have systemic toxicity in clinical animal studies [79,80]. In addition, a few FDA-approved drugs have also been found to have ERSA activity, such as celecoxib and bortezomib, although this activity was not discovered until after the main pharmacological action had been approved by the FDA [45,81,82]. In conclusion, there is still a long way to go for ER stress to establish a clinical treatment effect.

On the other hand, autophagy is also an important regulatory factor in the occurrence and development of cancer. Apoptosis and autophagy interact in both normal physiology and a wide range of diseases, and are crucial in determining the fate of cells [48,57]. Up to now, there is no research report that autophagy occurs in cancer cells after TDA treatment. In this study, we found that after TDA treatment of colon cancer cells, LC3-I conversion to LC3-II increased, the expression of P62 decreased and the number of autophagosomes also showed an increase as determined by immunofluorescence staining, indicating that TDA induced autophagy. Autophagy removes damaged organelles through lysosomal degradation and retrievals components to meet cellular metabolic needs [47]. Therefore, autophagy is essential for homeostasis and mediates resistance to radiation, chemotherapy and some targeted therapies [83]. More and more evidence show that autophagy inhibitors such as bafilomycin A1 and chloroquine (CQ) can enhance the effects of different cancer treatments, and clinical trials have been initiated [84]. However, the role of autophagy in cancer remains controversial. Some studies have suggested that cancer cells develop autophagy as a temporary survival mechanism after antitumor drug therapy [85,86]. Oppositely, some treatments can induce autophagic death or both apoptosis and autophagic death [87,88]. Thus, the exact role of autophagy appears to be highly dependent on cell type and environment. In order to further study the interaction between autophagy and apoptosis under TDA treatment, we pretreated HCT116 cells with autophagy inhibitors. HCT116 cells are more sensitive to TDA treatment, and the proportion of apoptotic cells increases. It shows that TDA-induced autophagy is cytoprotective. Therefore, joint treatment with autophagy inhibitors can make TDA achieve the best therapeutic effect.

In recent years, many studies have shown that ER stress can act as an upstream target of autophagy. The molecular mechanisms of ER stress induced autophagy include UPR, Ca^2+^ imbalance and Bcl-2 [89,90,91]. After we pretreated cells with ER stress inhibitors, the level of TDA-induced autophagy decreased, suggesting that TDA-induced autophagy was most likely to occur through ER stress, but the exact mechanism has still not been revealed. Clearly, more research is needed to determine the role of autophagy in TDA-treated cancer cells.

## 4. Materials and Methods 

### 4.1. Animal Study

Animal experiments were conducted under the guidance of Animal Management Regulations in Chongqing University. In vivo tumorigenesis assays were conducted as previously described [92]. When the tumor volume reached about 50 mm^3^, mice were randomly divided into four groups and subcutaneously injected with TDA, TDA/4-PBA, TDA/CQ or PBS every two days. Tumor volume was measured every five days. The mice were sacrificed after twenty days of treatment, and tumors were harvested.

### 4.2. Cell Culture and Reagents

Human colon cancer cell lines (HCT116 and DLD1) were purchased from Procell Company (Wuhan, China) and human colon mucosal epithelial cell line (NCM460) were purchased from Fenghui Biotechnology (Hunan, China). All cells were cultured in DMEM containing 10% fetal bovine serum (FBS) and 100 U/mL mixture of penicillin and streptomycin. The TDA is provided by the School of Pharmacy, Chongqing University. Cell counting kit 8 was purchased from MCE. Annexin V-FITC/PI Apoptosis Detection kit was purchased from CWBIO (Beijing, China). Primary antibodies for Western blotting, immunofluorescence and immunohistochemistry against target proteins are shown as follows: Caspase9/P35/P10 (Proteintech, Chicago, IL, USA, 66169), Caspase3 (Zen Bioscience, Chengdu, China, 300968), cleaved-Caspase3 (Zen Bioscience, 380169), PARP1 (Zen Bioscience, 380451), LC3 (Sangon Biotech, Shanghai, China, D163557), ATG5 (Sangon Biotech, D121650),SQSTM1/p62 (Beyotime, Shanghai, China, AF5312), β-actin (Santa Cruz, sc-47778), cyclinD1 (Cell Signaling Technology, USA, 2978S), cyclinB1 (Cell Signaling Technology, 4135S), PERK (Cell Signaling Technology, 3192S), IRE1 (Zen Bioscience, 220399), XBP1(Zen Bioscience, 381710), CHOP (Zen Bioscience, 381679) and HSPA5 (Sangon Biotech, D260466).

### 4.3. Cell Viability and Colony Formation Assay 

Cell viabilities were assessed by cell counting kit 8 (CCK8) assay. Cells were seeded in 96-well plates at a concentration of 5 × 10^3^ per well. After the cells adhered to the wall, the cells were treated with different concentrations of TDA and cultured for 48 h. Then, CCK8 was added and incubated at 37 °C for 2 h and absorbance value was measured at 450 nm of wavelength. Colony-formation assay was conducted as previously described [93]. Colonies with more than 50 cells were counted.

### 4.4. Flow Cytometric Analysis

HCT116 and DLD1 cells were seeded into a 6-well plate, attached overnight and were treated with TDA for 24 h. Then, cells were collected and stained with Annexin V-FITC/PI apoptosis detection kit and subjected to an apoptosis assay. Cell cycle analysis were conducted as previously described [94], cells were incubated with indicated concentration of TDA for 24 h, cells were rinsed in precooled PBS and followed by fixed with 75% alcohol overnight at 4 °C. Then cells were digested with 1% RNase A and stained with 1 mg/mL PI for 30 min. All of these experiments were performed according to the corresponding manufacturer’s instructions. Experimental data were analyzed using Flow Jo VX (Becton, Dickinson and Company, Franklin Lake, NJ, USA) and ModiFit LT 4.1 (Verity Software House, Topsham, ME, USA).

### 4.5. Western Blotting Analysis

Protein extraction and Western blot analysis were conducted as previously depicted [93]. Cells were rinsed in precooled PBS and proteins were extracted using RIPA lysis buffer (150 mM NaCl, 50 mM Tris-HCl, pH 8, 0.5% sodium deoxycholate, 1% NP-40, 0.2% SDS) containing protease inhibitor cocktail (EDTA free) (MedChemExpress, HY-K0011). After centrifugation (13,000× *g*, 10 min), total proteins were quantified with BCA Protein Assay Kit (CWBIO, Beijing, China, CW0014). Equal amounts of protein samples (30−80 μg) were separated on SDS-PAGE gels and then transferred to 0.45 μm PVDF membranes (GE Healthcare, Erlangen, Germany, A29280264). After incubation of the membranes with primary antibodies (1:1000) at 4 °C overnight, the samples were incubated with the secondary antibodies (1:5000) conjugated with horseradish peroxidase for 1 h at room temperature. The blots were visualized with WesternBright^TM^ ECL reagent (advansta, Bering Dr, San Jose, CA, USA, 191026-11) using ChemiDoc^TM^ XRS+ (Bio Rad, Berkeley, CA, USA).

### 4.6. Real-Time PCR Analysis

Total RNA was extracted using trizol reagent as described [93], quantitative real-time PCR was performed using SYBR-Green qPCR master mix. β-actin was used to normalize sample. The RT-PCR primers were used as follows: HSPA5, forward: CTGTCCAGGCTGGTGTGCTCT, reverse: CTTGGTAGGCACCACTGTGTTC, PERK, forward: GTCCCAAGGCTTTGGAATCTGTC, reverse: CCTACCAAGACAGGAGTTCTGG, IRE1, forward: CACCTCCACTCCCTCAACAT, reverse: CTTCTTGCAGAGGCCAAAGT, ATF6, forward: CAGACAGTACCAACGCTTATGCC, reverse: GCAGAACTCCAGGTGCTTGAAG, XBP1, forward: GTTGGGCATTCTGGACAACT, reverse: AAGGGAGGCTGGTAAGGAAC, ATF4, forward: TTCTCCAGCGACAAGGCTAAGG, reverse: CTCCAACATCCAATCTGTCCCG, CHOP, forward: CAGAACCAGAGGTCACA, reverse: AGCTGTGCCACTTTCCTTTC, LC3, forward: GAGAAGCAGCTTCCTGTTCTGG, reverse: GTGTCCGTTCACCAACAGGAAG, ATG5, forward: CCCTCTTGGGGTACATGTCT, reverse: CGTCCAAACCACACATCTCG, β-actin, forward: CCTAGAAGCATTTGCGGTGG, reverse: GAGCTACGAGCTGCCTGACG.

### 4.7. Confocal Microscopy Imaging 

To evaluate the expression level of endogenous LC3, we treated cells with TDA for 24 h, and then fixed with 4% paraformaldehyde and permeabilized with 0.2% Triton X-100, followed by blocking with 10% goat serum, cells were incubated with anti-LC3 antibody (1:200) and Alexa Fluor 555 goat anti-mouse IgG (H + L) antibody, then images were visualized under confocal microscopy [92].

### 4.8. Immunohistochemistry

Tumors obtained from HCT116 xenograft mice were fixed with 4% paraformaldehyde, embedded in paraffin and sectioned. Tumor slides were incubated overnight with the primary antibody at 4 °C and then incubated with a secondary antibody, and the chromogenic reaction was conducted with 3,3-diaminobenzidine and counterstained with hematoxylin.

### 4.9. Statistical Analysis

A two-tailed Student’s paired *t*-test and one-way ANOVA were used for statistical analysis of experimental data and data were expressed as mean ± SEM of at least three independent experiments. A value of *p* < 0.05 was considered statistically significant.

## 5. Conclusions

In summary, our results indicate that TDA may be a promising anticancer drug for the treatment of colon cancer, and excessive endoplasmic reticulum stress may be a key molecular event in the anticancer effect of TDA. TDA-induced ER stress leads to apoptosis through the IRE1/XBP1 pathway and the PERK/CHOP pathway. Interestingly, TDA-induced ER stress also triggered autophagy, which plays a protective role in response to ER stress, while the combination of autophagy inhibitors can significantly enhance the anticancer effect of TDA on colon cancer cells. These findings provide new insights into the mechanisms by which TDA inhibits colon cancer and support clinical studies targeting endoplasmic reticulum stress pathways, and highlighting the role of autophagy inhibitors in optimizing cancer treatment.

## Data Availability

Not applicable.

## Supplementary Materials

The following are available online at https://www.mdpi.com/article/10.3390/ijms22115566/s1.

## Abbreviations

CRC	Colorectal cancer
ER	Endoplasmic Reticulum
UPR	Unfolded protein response
HSPA5	Heat shock protein A5
IRE1α	Inositol-requiring enzyme 1α
PERK	Protein kinase R-like ER kinase
ATF6	Activating transcription factor 6
XBP1	X-box binding protein 1
CHOP	C/EBP homologous protein
ATF4	Activating transcription factor 4
eIF2α	Eukaryotic translation initiation factor 2α
PARP1	Poly (ADP-ribose) polymerase family, member 1
Caspase9/3	Cysteinyl aspartate specific proteinase 9/3
JNK	The c-Jun N-terminal kinases
TRAF2	Tumor necrosis factor alpha (TNFa) receptor associated factor 2
mTOR	The mammalian target of rapamycin
PI3K	Phosphatidylinositol 3 kinase
AKT	Protein kinase B
MET	Hepatocyte growth factor receptor
LC3B	Microtubule-associated protein 1 light chain 3
ATG5	Autophagy related 5
P62	Sequestosome 1
4-PBA	4-phenylbutyrate
CQ	Chloroquine
3-MA	3-methyladenine
CCK-8	Cell Counting Kit-8
