# Supplementary material for: Autophagy Induction by Trichodermic Acid Attenuates Endoplasmic Reticulum Stress-Mediated Apoptosis in Colon Cancer Cells
Source: Int J Mol Sci. 2021 May 25;22(11):5566. doi: 10.3390/ijms22115566 (PMC8197497; doi:10.3390/ijms22115566)
Supplement: Supplementary file 1 [file ijms-22-05566-s001.zip › ijms-1203672-supplementary.pdf]

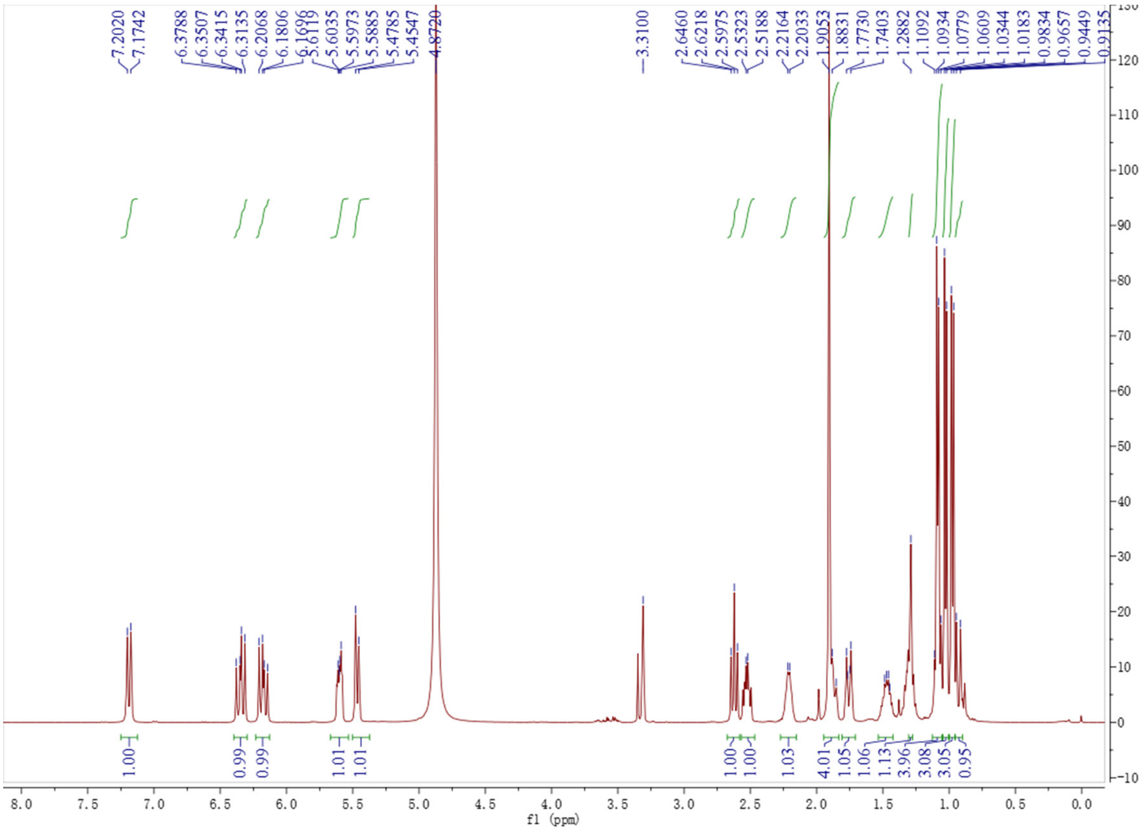

Figure S1. ^{1}H NMR spectrum of **trichodermic acid** (CD_3OD, 400 MHz).

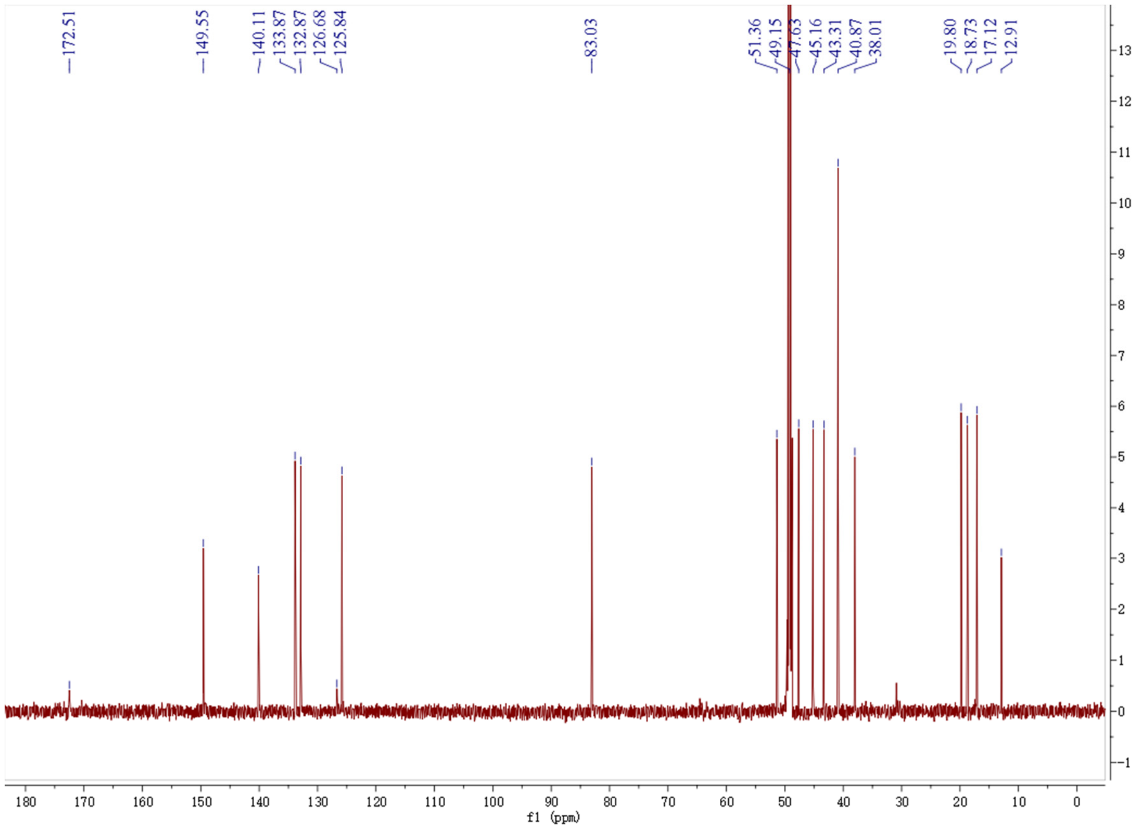

Figure S2. ^{13}C NMR spectrum of **trichodermic acid** (CD_3OD, 100 MHz).

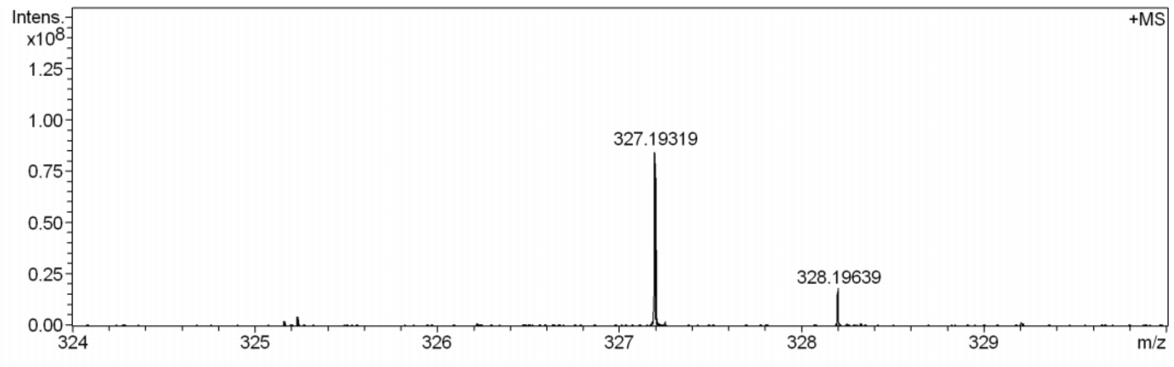

Meas. m/z	#	Formula	Score	m/z	err [ppm]	Mean err [ppm]	mSigma	rdb	e⁻ Conf	N-Rule
327.19319	1	C 19 H 28 Na O 3	100.00	327.19307	-0.37	-0.30	6.2	5.5	even	ok

Figure S3. HRESIMS spectrum of trichodermic acid.

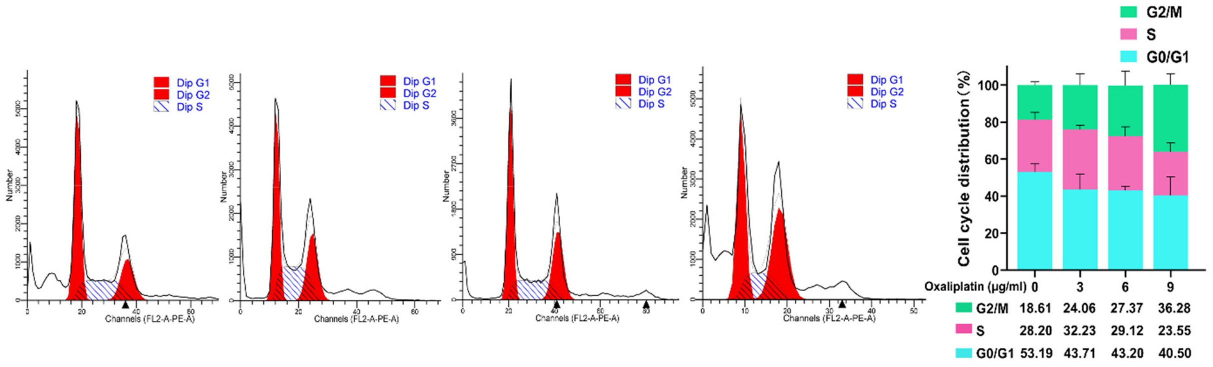

Figure S4. Flow cytometric analysis of cell cycle in HCT116 cells upon oxaliplatin treatment as indicated concentration for 24h was performed.
